# Venereal Disease in Scotland

**Published:** 1917-07-07

**Authors:** 


### VENEREAL DISEASE IN SCOTLAND.

### The Trend of Progress in Treatment.

LOCAL authorities in Scotland have, it would appear, made but slow progress in the organisation of schemes for the treatment of venereal diseases. In a circular dated June 1, 1917, the Local Government Board for Scotland invites those authorities which have not already done so to report upon the steps which they are taking in the matter, and makes for their guidance some observations "on certain points which may be engaging their attention." These points are of interest because they have, in many cases, arisen in England either in the preparation of schemes or in practice during the few months' working of schemes adopted and approved early in the year. The Local Government Board for Scotland is not only making good use of experience gained elsewhere; the "necessity for uniformity between England and Scotland" is plainly mentioned.

Some of the points raised are well deserving of attention. (1) The *salvarsan substitutes* at present approved are kharsivan, arsenobillon, novarsenobillon, diarsenol, and galyl. The use of the two last-named preparations has now been approved in England. Galyl had become so well known and had been so widely used that its omission from the first list of approved substitutes caused some surprise, and a demand for its inclusion was inevitable. (2) "*Chronic Cases.*"—Inquiries have been made as to whether the facilities provided are applicable to remote syphilitic manifestations, tertiary lesions, locomotor ataxia, etc. The Board has adopted the view that, for the present, schemes for diagnosis, treatment, etc., should apply only to venereal diseases during their communicable stages, which is clearly the intention of the Regulations. It is greatly to be regretted that no provision is made with regard to cases of congenital syphilis, which afford a wide and much neglected field for most valuable treatment and pathological inquiry. (3) *Pathological Specimens and Outfits.*—It has been suggested that fees should be payable to medical practitioners by local authorities for the taking of specimens for examination; the Board considers that, "for the present at least," payment of such fees should not be made, but that in exceptional cases the medical officer of health should make arrangements for a "competent person" to visit the patient at his home for the purpose of taking the specimen. It is to be presumed, and to be hoped, that the "competent person" is to be a qualified medical man; the employment of laboratory assistants for such work being open to grave objection, however capable they may be of performing it. It is especially in obtaining specimens of blood for the Wassermann reaction that difficulty is anticipated, and, as the Board points out also with regard to the question of treatment of patients in their homes, the "normal course" is for the patient to attend at a clinic or treatment centre, where everything necessary is provided free of charge. The medical officer of a treatment centre, or some other approved specialist, where considerations of time and distance allow, may visit a patient at his home, but the Board for Scotland will not allow grants, either in payment to general medical practitioners for home treatment, or in payment for drugs used other than approved salvarsan substitutes. The necessary outfits for taking material for pathological examination are, of course, to be always supplied to medical practitioners free of charge, and the Board agrees that the cost of postage to the laboratory should be defrayed. The Local Medical Committee for the County of London, when reporting in April last after consideration of the provisional scheme adopted by the Councils of London and the adjoining counties, called attention to some "defects" in the scheme and made certain suggestions. The committee advised that a special application on each occasion, upon Form V. 1., for a pathological outfit is unnecessary, as a new one should be sent out from the laboratory to the practitioner whenever one has been used by him. The same committee took exception to the request that certain particulars should be given by the practitioner when sending a specimen to the laboratory (Form V. 4), objecting that the pathologist need not, and should not, be informed concerning the patient's symptoms, the treatment which has been adopted, or the results of previous pathological examinations. "The requirement of this information cannot fail to raise a suspicion either that the pathologist is not capable of making the investigation, or that the investigation itself is of an indefinite and untrustworthy nature." Undoubtedly further light will be thrown upon the reliability and significance of the Wassermann reaction, just as experience will bring more nearly into line the different (and at present widely differing) schools in the matter of "salvarsan" treatment.

The question of the payment of the travelling expenses of patients to and from a treatment centre has arisen in Scotland, as elsewhere, and the Board has issued a memorandum on the subject (Form V. 12). The gist of this is that the Board will allow the inclusion, as part of the cost of the scheme of a local authority, of such expenditure "within reasonable limits": the medical officer of health is made responsible for limiting the payments to cases of necessity, for securing secrecy, etc. The Board "had some doubt whether travelling expenses should be repaid to any patients, and has only been willing to assent to the arrangements as an experiment."

The forms for records to be kept and returns to be made by institutions approved by the Board for Scotland are practically identical with those adopted in England; as also are the various forms, which are reproduced even as regards colour of paper. There are points upon which criticism of these might be offered, but it is to be remembered that the schemes for the diagnosis and treatment of venereal diseases were prepared somewhat hastily and under conditions of unusual difficulty. These schemes are tentative, and the provisional arrangements made under them are subject to revision as the result of the experience gained during the first year of working. Under adverse circumstances much has already been accomplished, and both the Local Government Boards and the Health Authorities concerned are deserving of congratulation upon the success attained.

